# Supplementary material for: Incidence of frailty-related fracture among Medicaid beneficiaries living with HIV and cancer: A cohort study
Source: PLoS One. 2026 May 21;21(5):e0348898. doi: 10.1371/journal.pone.0348898 (PMC13193461; doi:10.1371/journal.pone.0348898)
Supplement: S1 Table — (DOCX) [file pone.0348898.s001.docx]

| Table S1. The International Classification of Diseases 9^th^ edition (ICD-9) codes used to identify HIV diagnoses, cancer diagnoses and fracture diagnosis. | |
| --- | --- |
| Variable | ICD-9^a^ |
| Human Immunodeficiency Virus | 042-044, 079.53, 795.71, V08 |
| AIDS-defining cancers |  |
| Cervical | 180.X |
| Kaposi’s sarcoma | 176.X |
| Non-Hodgkin’s lymphoma | 200.X, 202.X |
| Non-AIDS-defining cancers |  |
| Anal^b^ | 154.2, 154.3 |
| Bladder | 188.X |
| Brain | 191.X |
| Breast | 174.X, 175.X |
| Colon | 153.X |
| Esophagus | 150.X |
| Head & neck | 140.X-149.X, 160.X, 161.X |
| Hodgkin’s lymphoma^b^ | 201.X |
| Kidney | 189.X |
| Larynx | 161.X |
| Leukemia | 204.X-208.X |
| Liver^b^ | 155.X |
| Lung | 162.2-162.5, 162.8, 162.9 |
| Melanoma | 172.X |
| Myeloma | 203.X |
| Oropharynx^b^ | 146.X |
| Ovary | 183.X |
| Pancreas | 157.X |
| Penile^b^ | 187.X |
| Prostate | 185.X |
| Rectal | 154.0, 154.1 |
| Stomach^b^ | 151.X |
| Uterine | 179.X, 182.X |
| Vaginal/vulvar^b^ | 184.X |
| Frailty-related fractures |  |
| Hip fracture | 733.14, 820.X |
| Vertebral | 805.X |
| Pelvic | 808.X |
| Wrist | 813.X |
| Note: ^a^A code ending with “.X” indicates a wildcard. Any number could appear after the decimal place.  ^b^Infection-related non-AIDS-defining cancer. In addition to oropharyngeal cancers, head-neck cancers with a code 141.0, 141.6, and 149.1 were classified as infection-related. | |
