# Supplementary material for: Incidence of frailty-related fracture among Medicaid beneficiaries living with HIV and cancer: A cohort study
Source: PLoS One. 2026 May 21;21(5):e0348898. doi: 10.1371/journal.pone.0348898 (PMC13193461; doi:10.1371/journal.pone.0348898)
Supplement: S2 Table — (DOCX) [file pone.0348898.s002.docx]

| Table S2. Baseline characteristics of Medicaid beneficiaries by HIV status, 2001-2015 | | |
| --- | --- | --- |
| Characteristics | No HIV | HIV |
| N | 14,554,711 | 159,188 |
| Age (year) (Median (Q25, Q75)) | 43.5 (35.2, 53.8) | 43.9 (37.4, 51.0) |
| Female, N (%) | 8,314,883 (57.1%) | 55,316 (34.7%) |
| Race/ethnicity, N (%) |  |  |
| non-Hispanic white | 6,188,473 (42.5%) | 39,447 (24.8%) |
| non-Hispanic Black | 2,999,662 (20.6%) | 76,046 (47.8%) |
| Hispanic | 2230468 (15.3%) | 14892 (9.4%) |
| Other/Missing | 3136108 (21.5%) | 28803 (18.1%) |
| State, N (%) |  |  |
| Alabama | 186,581 (1.3%) | 1,694 (1.1%) |
| California | 4,118,455 (28.3%) | 26,724 (16.8%) |
| Colorado | 206,996 (1.4%) | 713 (0.4%) |
| Florida | 922,304 (6.3%) | 18,551 (11.7%) |
| Georgia | 445,851 (3.1%) | 6,799 (4.3%) |
| Illinois | 1,196,744 (8.2%) | 8,595 (5.4%) |
| Massachusetts | 748,713 (5.1%) | 6,858 (4.3%) |
| Maryland | 389,126 (2.7%) | 6,631 (4.2%) |
| North Carolina | 451,637 (3.1%) | 6,228 (3.9%) |
| New York | 2,482,128 (17.1%) | 56,134 (35.3%) |
| Ohio | 930,941 (6.4%) | 4,321 (2.7%) |
| Pennsylvania | 860,089 (5.9%) | 4,391 (2.8%) |
| Texas | 842,897 (5.8%) | 7,971 (5.0%) |
| Washington | 772,249 (5.3%) | 3,578 (2.2%) |
| Note: HIV, human immunodeficiency virus; SD, standard deviation; N, number; ^a^Other included American Indian or Alaska native, Asian, Native Hawaiian or other Pacific Islander, more than one race, without indication of Hispanic ethnicity. | | |
