# Supplementary material for: Incidence of frailty-related fracture among Medicaid beneficiaries living with HIV and cancer: A cohort study
Source: PLoS One. 2026 May 21;21(5):e0348898. doi: 10.1371/journal.pone.0348898 (PMC13193461; doi:10.1371/journal.pone.0348898)
Supplement: S3 Table — (DOCX) [file pone.0348898.s003.docx]

| Table S3. Crude incidence rates of first frailty-related fracture and death by HIV and Non-AIDS defining cancer (NADC) status and age | | | | | | | | |
| --- | --- | --- | --- | --- | --- | --- | --- | --- |
| Age Group | Both HIV and NADC | | Only HIV | | Only NADC | | No HIV and NADC | |
|  | Events/PYs | IR (95% CI) | Events/PYs | IR (95% CI) | Events/PYs | IR (95% CI) | Events/PYs | IR (95% CI) |
| 30-44 |  |  |  |  |  |  |  |  |
| Fracture | 60/  4,836 | 1.24  (0.96, 1.59) | 2,048/  276,583 | 0.74  (0.71, 0.77) | 670/  67,705 | 0.99  (0.92, 1.07) | 57,125/  1,4076,918 | 0.41  (0.40, 0.41) |
| Death | 399/  4,836 | 8.25  (7.47, 9.09) | 4,810/  276,583 | 1.74  (1.69, 1.79) | 1,802/  67,705 | 2.66  (2.54, 2.79) | 23,035/  14,076,918 | 0.16  (0.16, 0.17) |
| 45-49 |  |  |  |  |  |  |  |  |
| Fracture | 77/  4,717 | 1.63  (1.30, 2.03) | 1,468/  149,156 | 0.98  (0.93, 1.04) | 842/  62,027 | 1.36  (1.27, 1.45) | 27,158/  4,448,093 | 0.61  (0.60, 0.62) |
| Death | 409/  4,717 | 8.67  (7.86, 9.54) | 2,774/  149,156 | 1.86  (1.79, 1.93) | 2,455/  62,027 | 3.96  (3.80, 4.12) | 17,561/  4,448,093 | 0.39  (0.39, 0.40) |
| 50-54 |  |  |  |  |  |  |  |  |
| Fracture | 134/  5,905 | 2.27  (1.91, 2.68) | 1,580/  128,949 | 1.23  (1.17, 1.29) | 1,696/  96,220 | 1.76  (1.68, 1.85) | 34,517/  4,307,206 | 0.80  (0.79, 0.81) |
| Death | 508/  5,905 | 8.60  (7.88, 9.38) | 2,433/  128,949 | 1.89  (1.81, 1.96) | 4,932/  96,220 | 5.13  (4.98, 5.27) | 25,084/  4,307,206 | 0.58  (0.58, 0.59) |
| 55-59 |  |  |  |  |  |  |  |  |
| Fracture | 128/  5,434 | 2.36  (1.97, 2.79) | 1,159/  86,418 | 1.34  (1.27, 1.42) | 2,442/  129,940 | 1.88  (1.81, 1.95) | 37,730/  4,026,119 | 0.94  (0.93, 0.95) |
| Death | 502/  5,434 | 9.24  (8.46, 10.07) | 1,671/  86,418 | 1.93  (1.84, 2.03) | 7,952/  129,940 | 6.12  (5.99, 6.26) | 30,421/  4,026,119 | 0.76  (0.75, 0.76) |
| 60-64 |  |  |  |  |  |  |  |  |
| Fracture | 65/  3,087 | 2.11  (1.64, 2.67) | 728/  49,362 | 1.47  (1.37, 1.58) | 2,712/  125,577 | 2.16  (2.08, 2.24) | 37,759/  3,609,120 | 1.05  (1.04, 1.06) |
| Death | 380/  3,087 | 12.31  (11.12, 13.60) | 1,001/  49,362 | 2.03  (1.91, 2.16) | 10,303/  125,577 | 8.20  (8.05, 8.36) | 31,546/  3,609,120 | 0.87  (0.86, 0.88) |
| IR, incidence rate per 100 person-years; PY, person-year. | | | | | | | | |
