# Supplementary material for: Incidence of frailty-related fracture among Medicaid beneficiaries living with HIV and cancer: A cohort study
Source: PLoS One. 2026 May 21;21(5):e0348898. doi: 10.1371/journal.pone.0348898 (PMC13193461; doi:10.1371/journal.pone.0348898)
Supplement: S4 Table — (DOCX) [file pone.0348898.s004.docx]

| Table S4. Crude incidence rates of first frailty-related fracture and death by HIV and Non-AIDS defining cancer (NADC) status, age, and sex | | | | | | | | |
| --- | --- | --- | --- | --- | --- | --- | --- | --- |
| Age Group | Both HIV and NADC | | Only HIV | | Only NADC | | No HIV and NADC | |
|  | Events/PYs | IR (95% CI) | Events/PYs | IR (95% CI) | Events/PYs | IR (95% CI) | Events/PYs | IR (95% CI) |
| *Female* | | | | | | | | |
| 30-44 |  |  |  |  |  |  |  |  |
| Fracture | 29/2,663 | 1.09  (0.74, 1.54) | 858/133,202 | 0.64  (0.60, 0.69) | 462/51,277 | 0.90  (0.82, 0.99) | 32,427/9,108,129 | 0.36  (0.35, 0.36) |
| Death | 210/2,663 | 7.89  (6.87, 9.01) | 2,285/133,202 | 1.72  (1.65, 1.79) | 1,087/51,277 | 2.12  (2.00, 2.25) | 10,959/9,108,129 | 0.12  (0.12, 0.12) |
| 45-49 |  |  |  |  |  |  |  |  |
| Fracture | 33/2,441 | 1.35  (0.95, 1.88) | 631/64,809 | 0.97  (0.90, 1.05) | 585/45,385 | 1.29  (1.19, 1.40) | 15,255/2,632,110 | 0.58  (0.57, 0.59) |
| Death | 191/2,441 | 7.82  (6.77, 8.99) | 1,148/64,809 | 1.77  (1.67, 1.88) | 1,339/45,385 | 2.95  (2.80, 3.11) | 8,335/2,632,110 | 0.32  (0.31, 0.32) |
| 50-54 |  |  |  |  |  |  |  |  |
| Fracture | 59/2,855 | 2.07  (1.59, 2.65) | 671/52,425 | 1.28  (1.19, 1.38) | 1,115/64,482 | 1.73  (1.63, 1.83) | 20,417/2,460,542 | 0.83  (0.82, 0.84) |
| Death | 209/2,855 | 7.32  (6.38, 8.37) | 900/52,425 | 1.72  (1.61, 1.83) | 2,421/64,482 | 3.75  (3.61, 3.91) | 11,271/2,460,542 | 0.46  (0.45, 0.47) |
| 55-59 |  |  |  |  |  |  |  |  |
| Fracture | 66/2,399 | 2.75  (2.14, 3.48) | 491/33,344 | 1.47  (1.35, 1.61) | 1,516/79,738 | 1.90  (1.81, 2.00) | 23,473/2,297,761 | 1.02  (1.01, 1.03) |
| Death | 191/2,399 | 7.96  (6.89, 9.15) | 577/33,344 | 1.73  (1.59, 1.88) | 3,606/79,738 | 4.52  (4.38, 4.67) | 13,620/2,297,761 | 0.59  (0.58, 0.60) |
| 60-64 |  |  |  |  |  |  |  |  |
| Fracture | 37/1,178 | 3.14  (2.24, 4.28) | 334/19,386 | 1.72  (1.55, 1.92) | 1,650/72,044 | 2.29  (2.18, 2.40) | 25,192/2,146,867 | 1.17  (1.16, 1.19) |
| Death | 114/1,178 | 9.68  (8.02, 11.58) | 306/19,386 | 1.58  (1.41, 1.76) | 4,609/72,044 | 6.40  (6.21, 6.58) | 14,776/2,146,867 | 0.69  (0.68, 0.70) |
| *Male* | | | | | | | | |
| 30-44 |  |  |  |  |  |  |  |  |
| Fracture | 31/2,172 | 1.43  (0.99, 2.00) | 1,190/143,380 | 0.83  (0.78, 0.88) | 208/16,427 | 1.27  (1.10, 1.45) | 24,698/4,968,788 | 0.50  (0.49, 0.50) |
| Death | 189/2,172 | 8.70  (7.52, 10.01) | 2,525/143,380 | 1.76  (1.69, 1.83) | 715/16,427 | 4.35  (4.04, 4.68) | 12,076/4,968,788 | 0.24  (0.24, 0.25) |
| 45-49 |  |  |  |  |  |  |  |  |
| Fracture | 44/2,276 | 1.93  (1.42, 2.57) | 837/84,347 | 0.99  (0.93, 1.06) | 257/16,642 | 1.54  (1.36, 1.74) | 11,903/1,815,983 | 0.66  (0.64, 0.67) |
| Death | 218/2,276 | 9.58  (8.37, 10.91) | 1,626/84,347 | 1.93  (1.84, 2.02) | 1,116/16,642 | 6.71  (6.32, 7.11) | 9,226/1,815,983 | 0.51  (0.50, 0.52) |
| 50-54 |  |  |  |  |  |  |  |  |
| Fracture | 75/3,050 | 2.46  (1.95, 3.06) | 909/76,524 | 1.19  (1.11, 1.27) | 581/31,738 | 1.83  (1.69, 1.98) | 14,100/1,846,664 | 0.76  (0.75, 0.78) |
| Death | 299/3,050 | 9.80  (8.74, 10.96) | 1,533/76,524 | 2.00  (1.90, 2.11) | 2,511/31,738 | 7.91  (7.61, 8.23) | 13,813/1,846,664 | 0.75  (0.74, 0.76) |
| 55-59 |  |  |  |  |  |  |  |  |
| Fracture | 62/3,035 | 2.04  (1.58, 2.60) | 668/53,074 | 1.26  (1.17, 1.36) | 926/50,202 | 1.84  (1.73, 1.97) | 14,257/1,728,358 | 0.82  (0.81, 0.84) |
| Death | 311/3,035 | 10.25  (9.16, 11.44) | 1,094/53,074 | 2.06  (1.94, 2.19) | 4,346/50,202 | 8.66  (8.40, 8.92) | 16,801/1,728,358 | 0.97  (0.96, 0.99) |
| 60-64 |  |  |  |  |  |  |  |  |
| Fracture | 28/1,908 | 1.47  (0.99, 2.09) | 394/29,976 | 1.31  (1.19, 1.45) | 1,062/53,533 | 1.98  (1.87, 2.11) | 12,567/1,462,253 | 0.86  (0.84, 0.87) |
| Death | 266/1,908 | 13.94  (12.34, 15.69) | 695/29,976 | 2.32  (2.15, 2.50) | 5,694/53,533 | 10.64  (10.36, 10.92) | 16,770/1,462,253 | 1.15  (1.13, 1.16) |
| IR, incidence rate per 100 person-years; PY, person-year. | | | | | | | | |
