# Supplementary material for: Incidence of frailty-related fracture among Medicaid beneficiaries living with HIV and cancer: A cohort study
Source: PLoS One. 2026 May 21;21(5):e0348898. doi: 10.1371/journal.pone.0348898 (PMC13193461; doi:10.1371/journal.pone.0348898)
Supplement: S5 Table — (DOCX) [file pone.0348898.s005.docx]

| Table S5. Proportion (%) of frailty-related fractures by age, body location, sex, and HIV and Non-AIDS defining cancer (NADC) status | | | | |
| --- | --- | --- | --- | --- |
| Age Group | Proportion | | | |
|  | Both HIV and NADC | Only HIV | Only NADC | No HIV and NADC |
| *Female* | | | | |
| 30-44 |  |  |  |  |
| Hip | 48.28 | 17.81 | 27.29 | 11.23 |
| Pelvic | 13.79 | 8.96 | 9.59 | 7.33 |
| Vertebral | 10.34 | 24.97 | 32.41 | 31.29 |
| Wrist | 27.59 | 48.26 | 30.70 | 50.14 |
| 45-49 |  |  |  |  |
| Hip | 36.36 | 20.92 | 23.62 | 14.95 |
| Pelvic | 9.09 | 9.23 | 9.55 | 7.06 |
| Vertebral | 21.21 | 23.69 | 35.85 | 29.39 |
| Wrist | 33.33 | 46.15 | 30.99 | 48.59 |
| 50-54 |  |  |  |  |
| Hip | 25.40 | 22.77 | 25.68 | 17.09 |
| Pelvic | 9.52 | 8.76 | 7.77 | 6.76 |
| Vertebral | 17.46 | 22.92 | 32.14 | 27.68 |
| Wrist | 47.62 | 45.55 | 34.41 | 48.46 |
| 55-59 |  |  |  |  |
| Hip | 36.23 | 24.40 | 27.54 | 19.92 |
| Pelvic | 4.35 | 9.72 | 8.08 | 6.95 |
| Vertebral | 27.54 | 24.80 | 34.26 | 27.26 |
| Wrist | 31.88 | 41.07 | 30.12 | 45.86 |
| 60-64 |  |  |  |  |
| Hip | 28.95 | 27.38 | 32.13 | 22.97 |
| Pelvic | 15.79 | 9.80 | 8.21 | 7.16 |
| Vertebral | 21.05 | 26.51 | 35.62 | 27.92 |
| Wrist | 34.21 | 36.31 | 24.04 | 41.95 |
| *Male* | | | | |
| 30-44 |  |  |  |  |
| Hip | 29.03 | 20.20 | 28.44 | 14.53 |
| Pelvic | 0 | 9.04 | 6.16 | 7.52 |
| Vertebral | 38.71 | 24.35 | 35.55 | 32.59 |
| Wrist | 32.26 | 46.42 | 29.86 | 45.36 |
| 45-49 |  |  |  |  |
| Hip | 31.82 | 24.36 | 27.38 | 17.84 |
| Pelvic | 9.09 | 8.08 | 5.70 | 7.39 |
| Vertebral | 29.55 | 25.17 | 40.30 | 33.92 |
| Wrist | 29.55 | 42.38 | 26.62 | 40.85 |
| 50-54 |  |  |  |  |
| Hip | 35.06 | 25.16 | 31.50 | 21.91 |
| Pelvic | 3.90 | 7.71 | 6.67 | 7.52 |
| Vertebral | 37.66 | 25.05 | 37.17 | 34.59 |
| Wrist | 23.38 | 42.08 | 24.67 | 35.98 |
| 55-59 |  |  |  |  |
| Hip | 41.27 | 32.40 | 32.32 | 25.70 |
| Pelvic | 6.35 | 6.92 | 6.53 | 7.38 |
| Vertebral | 23.81 | 24.74 | 41.16 | 34.93 |
| Wrist | 28.57 | 35.94 | 20.00 | 31.99 |
| 60-64 |  |  |  |  |
| Hip | 35.71 | 39.70 | 36.44 | 30.73 |
| Pelvic | 3.57 | 5.21 | 8.31 | 7.50 |
| Vertebral | 39.29 | 26.30 | 38.45 | 33.50 |
| Wrist | 21.43 | 28.78 | 16.80 | 28.26 |
