# Supplementary material for: Incidence of frailty-related fracture among Medicaid beneficiaries living with HIV and cancer: A cohort study
Source: PLoS One. 2026 May 21;21(5):e0348898. doi: 10.1371/journal.pone.0348898 (PMC13193461; doi:10.1371/journal.pone.0348898)
Supplement: S1 Fig — A) Risk of first frailty-related fracture among beneficiaries with AIDS-defining cancer; B) Risk of death among beneficiaries with AIDS-defining cancer; C) Risk of first frailty-related fracture among female beneficiaries with breast cancer; D) Risk of death among female beneficiaries with breast cancer; E) Risk of first frailty-related fracture among beneficiaries with colon cancer; F) Risk of death among beneficiaries with colon cancer; G) Risk of first frailty-related fracture among beneficiaries with lung cancer; H) Risk of death among beneficiaries with lung cancer; I) Risk of first frailty-related fracture among male beneficiaries with prostate cancer; J) Risk of death among male beneficiaries with prostate cancer. (DOCX) [file pone.0348898.s006.docx]

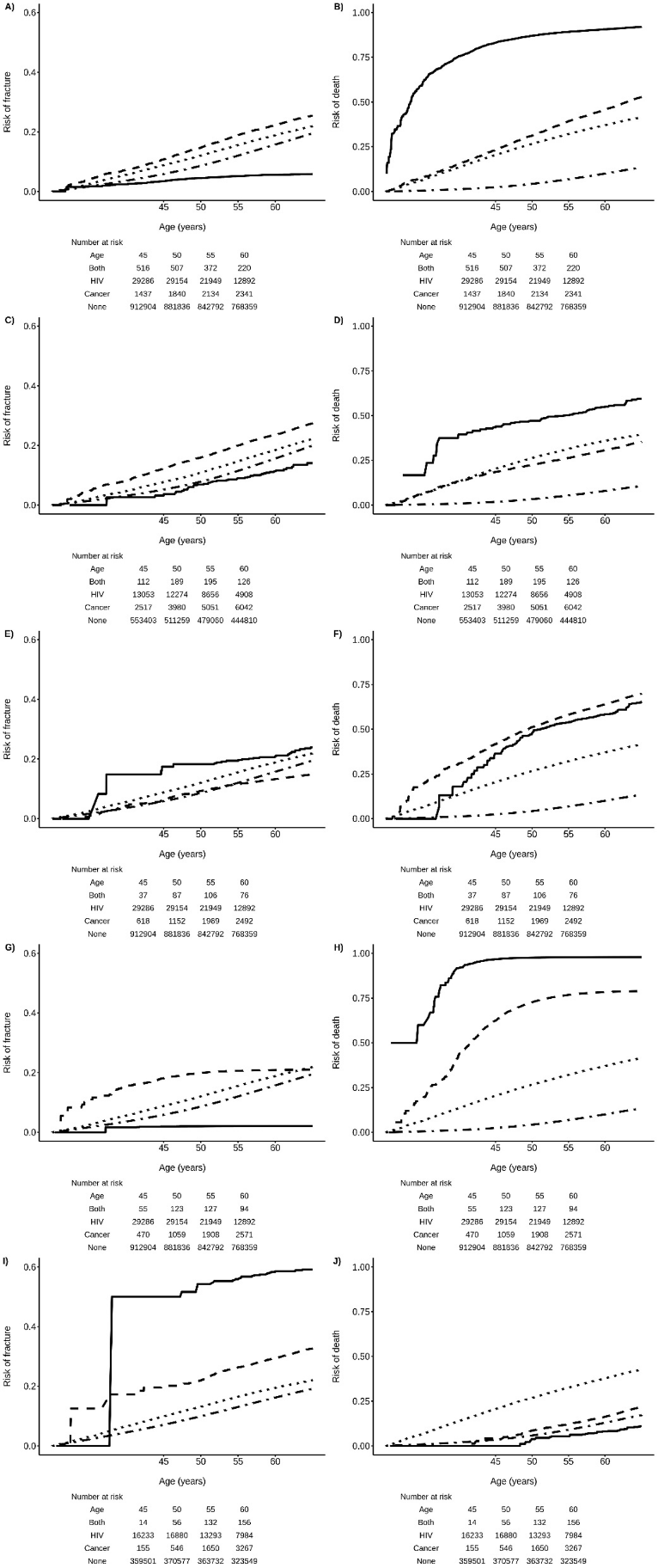


Figure S1. Cumulative incidence of frailty-related fracture and death by sex, age, and HIV and cancer type among Medicaid Beneficiaries, 2001-2015. A) Risk of first frailty-related fracture among beneficiaries with AIDS-defining cancer; B) Risk of death among beneficiaries with AIDS-defining cancer; C) Risk of first frailty-related fracture among female beneficiaries with breast cancer; D) Risk of death among female beneficiaries with breast cancer; E) Risk of first frailty-related fracture among beneficiaries with colon cancer; F) Risk of death among beneficiaries with colon cancer; G) Risk of first frailty-related fracture among beneficiaries with lung cancer; H) Risk of death among beneficiaries with lung cancer; I) Risk of first frailty-related fracture among male beneficiaries with prostate cancer; J) Risk of death among male beneficiaries with prostate cancer.
