# Supplementary material for: Incidence of frailty-related fracture among Medicaid beneficiaries living with HIV and cancer: A cohort study
Source: PLoS One. 2026 May 21;21(5):e0348898. doi: 10.1371/journal.pone.0348898 (PMC13193461; doi:10.1371/journal.pone.0348898)
Supplement: S2 Fig — A) Risk of first frailty-related fracture among beneficiaries in 2001–2005; B) Risk of death among beneficiaries in 2001–2005; C) Risk of first frailty-related fracture among female beneficiaries in 2006–2010; D) Risk of death among female beneficiaries in 2006–2010; E) Risk of first frailty-related fracture among beneficiaries in 2011–2015; F) Risk of death among beneficiaries in 2011–2015. (DOCX) [file pone.0348898.s007.docx]

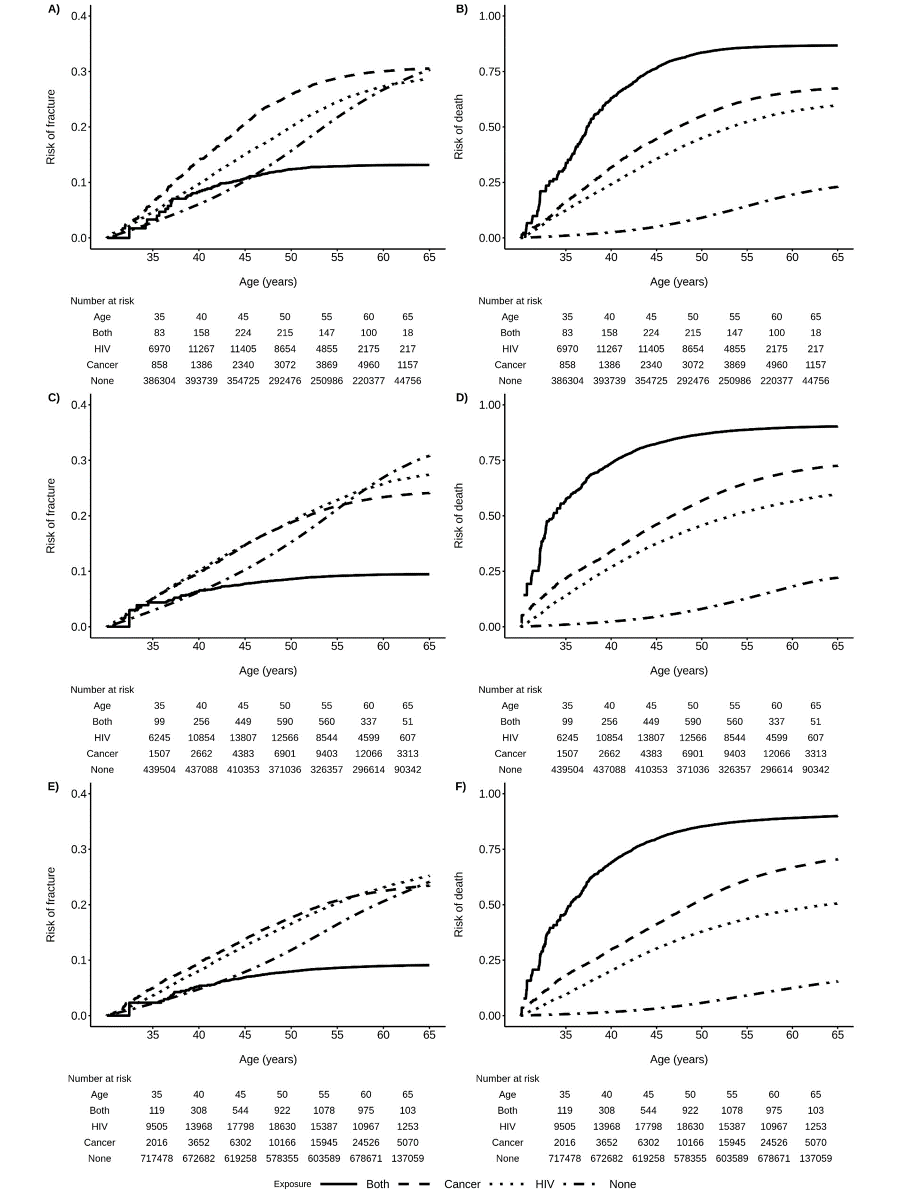


Figure S2. Cumulative incidence of frailty-related fracture and death by sex, age, HIV and Non-AIDS defining cancer, and calendar period among Medicaid Beneficiaries, 2001-2015. A) Risk of first frailty-related fracture among beneficiaries in 2001-2005; B) Risk of death among beneficiaries in 2001-2005; C) Risk of first frailty-related fracture among female beneficiaries in 2006-2010; D) Risk of death among female beneficiaries in 2006-2010; E) Risk of first frailty-related fracture among beneficiaries in 2011-2015; F) Risk of death among beneficiaries in 2011-2015.
